# Mixed-gas CH_4_/CO_2_/CO detection based on linear variable optical filter and thermopile detector array

**DOI:** 10.1186/s11671-019-3176-7

**Published:** 2019-11-25

**Authors:** Shaoda Zhang, Wu Bin, Binbin Xu, Xingyu Zheng, Binbin Chen, Xueqin Lv, Haisheng San, Werner Hofmann

**Affiliations:** 1 Pen-Tung Sah Institute of Micro-Nano Science and Technology, Xiamen University, Xiamen, 361005 China; 2 Shenzhen Research Institute of Xiamen University, Shenzhen, 518000 China; 3 Shenzhen MEMS-Frontier Electronics Co. Ltd., Shenzhen, 518107 China; 4 Institute of Solid State Physics, Technical University of Berlin, 10623 Berlin, Germany

**Keywords:** Linear variable optical filter, Tapered cavity, Multi-layer dielectrics, Thermopile detector, Mixed gas detectors

## Abstract

This paper presents the design, fabrication, and characterization of a middle-infrared (MIR) linear variable optical filter (LVOF) and thermopile detectors that will be used in a miniaturized mixed gas detector for CH_4_/CO_2_/CO measurement. The LVOF was designed as a tapered-cavity Fabry-Pérot optical filter, which can transform the MIR continuous spectrum into multiple narrow band-pass spectra with peak wavelength in linear variation. Multi-layer dielectric structures were used to fabricate the Bragg reflectors on the both sides of tapered cavity as well as the antireflective film combined with the function of out-of-band rejection. The uncooled thermopile detectors were designed and fabricated as a multiple-thermocouple suspension structure using micro-electro-mechanical system technology. Experimentally, the LVOF exhibits a mean full-width-at-half-maximum of 400 nm and mean peak transmittance of 70% at the wavelength range of 2.3~5 μm. The thermopile detectors exhibit a responsivity of 146 μV/°C at the condition of room temperature. It is demonstrated that the detectors can achieve the quantification and identification of CH_4_/CO_2_/CO mixed gas.

## Introduction

Gas sensors have a great demand in many industrial and real-life applications. In many of these applications, multiple gases must be monitored simultaneously over a long period of time with minimal maintenance and in different locations [1]. Taking natural gas for example, it contains a mixture of a large amount of methane (CH_4_) and small amount of various hydrocarbon gas (e.g., C_*x*_H_*y*_), which has emerged as a major energy source. However, when natural gas burns openly, the use of natural gas has been found to increase the risk of human health and environment. It produces a great deal of water vapor and a mixture of compounds, e.g., nitrogen oxides (N_2_O), carbon dioxide (CO_2_), and even carbon monoxide (CO) and fumes caused by the incomplete-combustion of natural gas [2]. Some toxic chemicals emitted by natural gas are not just harmful to residents, but the leaked natural gas also can cause an explosion. Over the last decades, the requirement for safety monitoring on natural gas and its combustion products is continuously increasing, resulting in a great amount of demand for the miniaturized mixed gas detectors [3]. The miniaturizations of gas detectors can bring about the low-cost and large-scale manufacturing processes as well as low power consumption. Meanwhile, it could also result in degraded analytical capabilities or reduced flexibility in multi-parameter measurement.

Gas detectors based on chemiresistive gas-sensing materials (e.g., metal-oxide semiconductors (MOSs), polymers, carbon nanotubes (CNTs), and moisture-absorbing materials) have been widely developed and applied due to its small size and low cost, but it is not satisfying because each detector detects only one type of gas with qualitative information regarding the gas concentration [4–7]. Moreover, the high operating temperature and the requirement for calibration and readjustment after a short period limit their application and increase the maintenance cost [7]. For these reasons, some gas analysis techniques have been developed for fabricating the miniaturized mixed gas sensors. Micro-gas chromatography (μGC) based on micro-electro mechanical systems (MEMS) technology has made a significant progress in recent decades [8]. A μGC system is a hybrid integration of several MEMS devices (e.g., injector, separation column, gas detector, micro-valves, and micro-pumps), which can provide an accurate analysis of complex gas mixtures [9, 10]. However, up to now, the handheld μGC instruments for on-site analysis are still not commercially available [8]. Optical sensing technique is another alternative solution for gas measurement [11, 12]. Fourier transform infrared (FTIR) spectrometer is a good example of an instrument that can measure mixed gas through analyzing specific spectral response in IR region. However, FTIR spectrometers are usually a bulky instrument, which is not suitable for gas monitoring due to its high cost and the lack of portability. MEMS-based scanning mirror (Michelson interferometer) is a recently emerging solution for the miniaturized FTIR spectrometers, which are capable of providing a set of continuously changing wavelengths across Near-IR (NIR) or Middle-IR (MIR) band [13–16]. However, the use of fast-response IR laser and detectors (e.g., the cooled PbSe or the HgCdTe photoconductive detector) will increase the cost and system size of spectrometer [15]. Another effective mixed gas measurement method based on IR absorption spectrum technology is the non-dispersive infrared (NDIR) gas detection, which can be realized by using multiple IR filter channels or using single gas channel with a spinning multi-filter chopper system [17]. Doubtlessly, both techniques will inevitably result in the increase of detector size and cost. For these reasons, many micro-optics devices have been used to construct the miniaturized NDIR multi-gas sensors, e.g., MEMS-based Fabry-Pérot (F-P) filters [18, 19], photonic crystal filters [20, 21], and linear variable optical filter (LVOF) [22, 23]

In this work, a miniaturized mixed gas (e.g., CH_4_/CO_2_/CO) detector based on NDIR gas detection mechanisms was fabricated using a MIR linear variable optical filter (LVOF) and MEMS-based uncooled thermopile detector array. The designs, fabrications, and characterizations of micro-devices and integrated gas detectors were presented in detail, respectively. The usages of these micro-devices make a compact integration of multiple gas detectors, which have significant advantages in small size as well as low cost and power consumption by using a light source, a gas cell, and a data process element when compared with the traditional NDIR gas detectors.

### Design and Experimental Methods

#### Design and Fabrication of LVOF

**Fig. 1:**
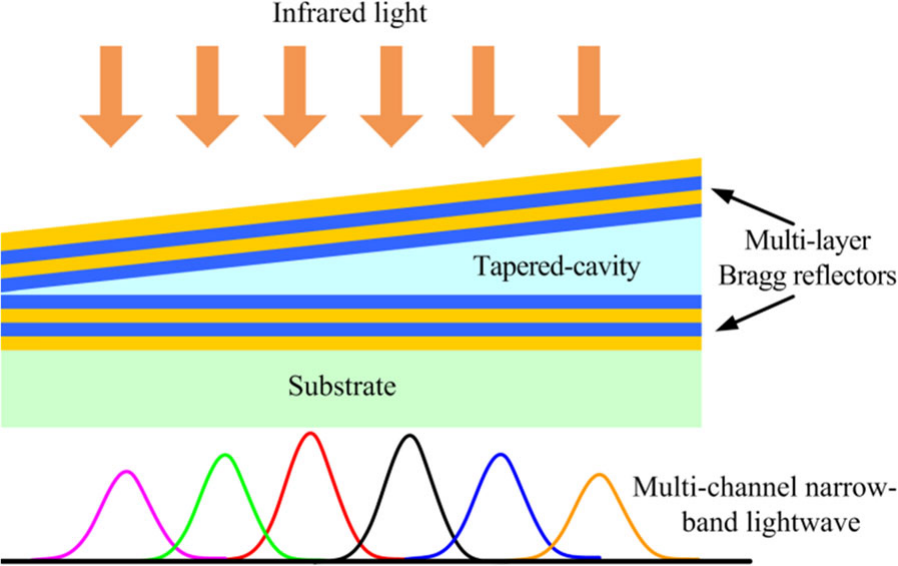
Schematic diagram of work princples of LVOF structure

As shown in Fig. 1, the LVOF is designed as a F-P type filter, consisting of a tapered cavity, two Bragg reflectors made respectively on both sides of tapered cavity, and a substrate. The cavity and the top reflector are continuously tapered with a linear variable thickness along the length of LVOF, resulting in a F-P type filter array structure with an infinite number of narrow pass-band filters placed side by side on substrate. As the MIR light is incident on the linear array of F-P type filters, the transmission light is band-pass filtered according to the width of each F-P cavity and thus by the spatial position along the length of the LVOF [18]. The thickness of each F-P cavity will determine the wavelength of the transmitted light at corresponding filter position. We focus on MIR band of 2.3~5.0 μm to design the LVOF configuration where most of characteristic gas absorption peaks (e.g., CO_2_, CO, N_2_O, and C_*x*_H_*y*_) related to indoor air quality and general industrial environment are centralized. Material selection is significantly important in optical filter design for achieving high transmissivity in targeted wavelength. Generally, the reflected films using metal layer have high absorptivity in infrared waveband, which will result in a round 15~30% of peak transmissivity in filter. By contrast, the reflectors using multi-layer dielectrics are able to create a higher peak transmissivity in filter, e.g., 60~70% in MIR band. In this work, a full dielectric multi-layer structure is considered to fabricate the reflectors of LVOF.

The reflectors on two side of the tapered cavity are designed as Bragg reflectors that are composed of alternate layers of high and low refractive index materials. The multi-layer structure and the high refractive index contrast can effectively increase the reflectivity of Bragg reflectors. The transmissivity of LVOF (*T*) can be calculated by [22]:

1 $$ T=\frac{T_0}{1+F{\left(\sin \theta \right)}^2}, $$

with

2 $$ {T}_0=\frac{\left(1-{R}_1\right)\left(1-{R}_2\right)}{{\left(1-\sqrt{R_1{R}_2}\right)}^2},\kern0.5em $$

3 $$ F=\frac{4\sqrt{R_1{R}_2}}{{\left(1-\sqrt{R_1{R}_2}\right)}^2}, $$

and

4 $$ \theta =\frac{1}{2}\left({\varphi}_1+{\varphi}_2-2\delta \right) $$

where *R*_1_ and *R*_2_ are the reflectivity of Bragg reflectors in up-side and down-side of tapered cavity, respectively. *φ*_1_ and *φ*_2_ are the phase change of reflected light in up-side and down-side Bragg reflectors, respectively. *δ* is the phase change of light, induced by the thickness of cavity layer *d*. As the incident light is normal (perpendicular) to the substrate, *δ* meets the following equation:

5 $$ 2\delta =2 knd=2\frac{2\pi }{\lambda } nd $$

where *n* is the refractive index of cavity layer. For a multi-layer Bragg reflector, the reflectivity and phase change of multi-layer dielectric film can be calculated by:

6 $$ R=\left(\frac{N_0-Y}{N_0+Y}\right)\;{\left(\frac{N_0-Y}{N_0+Y}\right)}^{\ast } $$

7 $$ \varphi =\mathrm{atan}\left[\frac{i{N}_0\left(Y-{Y}^{\ast}\right)}{{N_0}^2-Y{Y}^{\ast }}\right] $$

where *N*_0_ is the refractive index of incident dielectric layer, and *Y* is the admittance of multi-layer dielectric film, which can be expressed as *Y* = *C*/*B*. By means of matrix method, the characteristic matrix of a multi-layer dielectric film can be expressed as follows:

8 $$ \left[\begin{array}{c}\mathrm{B}\\ {}C\end{array}\right]=\prod \limits_{j=1}^k\left[\begin{array}{cc}\cos {\delta}_j& \frac{i}{\eta_j}\mathit{\sin}{\delta}_j\\ {}i{\eta}_j\mathit{\sin}{\delta}_j& \cos {\delta}_j\end{array}\right]\left[\begin{array}{c}1\\ {}{\eta}_{k+1}\end{array}\right] $$

where, *η*_*j*_ and *δ*_*j*_ are the admittance and phase change of *j*th dielectric layer, respectively. *η*_*j*_ = *N*_*j*_ and *δ*_*j*_ = 2*π N*_*j*_ *d*_*j*_ /*λ*. The peak wavelength (*λ*_*0*_) with maximum transmissivity can be calculated by:

9 $$ {\displaystyle \begin{array}{l}{\theta}_0=\frac{1}{2}\left({\varphi}_1+{\varphi}_2-2\delta \right)=\kern0.4em \frac{1}{2}\left({\varphi}_1+{\varphi}_2-2\frac{2\pi }{\lambda } nd\right)\\ {}\kern0.3em =\kern0.4em - k\pi \kern0.50em \left(\ k=0,1,2,\dots ..\right)\end{array}} $$

10 $$ {\uplambda}_0=\frac{2\mathrm{nd}}{k+\left[\frac{\varphi_1+{\varphi}_2}{2\pi}\right]}=\frac{2 nd}{m} $$

where *m* = *k* + (*φ*_1_ + *φ*_2_)/2π. From Eq. (10), it can be seen that the peak wavelength is a linear dependence on the thickness of cavity.

In this study, Si and SiO_2_ were selected as high and low refractive index materials, and the SiO_2_ was used to fabricate tapered cavity. The Si was used as substrate material. These materials are transparent in the MIR band, and they are MEMS compatible in fabrication process. The refractive index of Si and SiO_2_ is 3.43 and 1.42 in the wavelength range of 2.3~5.0 μm, respectively. The layer configuration of LVOF was designed as Si/(*LH*)^*n*^(*xL*)(*HL*)^*n*^*H*/Air, where *H* and *L* represent high and low refractive index layer, respectively, *n* is the number of *LH* pairs, and *x* is the changing factor of cavity thickness. It is noted that the reflectors will obtain the maximum reflectivity when the outmost layer of reflectors uses the high refractive index of Si material.

**Fig. 2:**
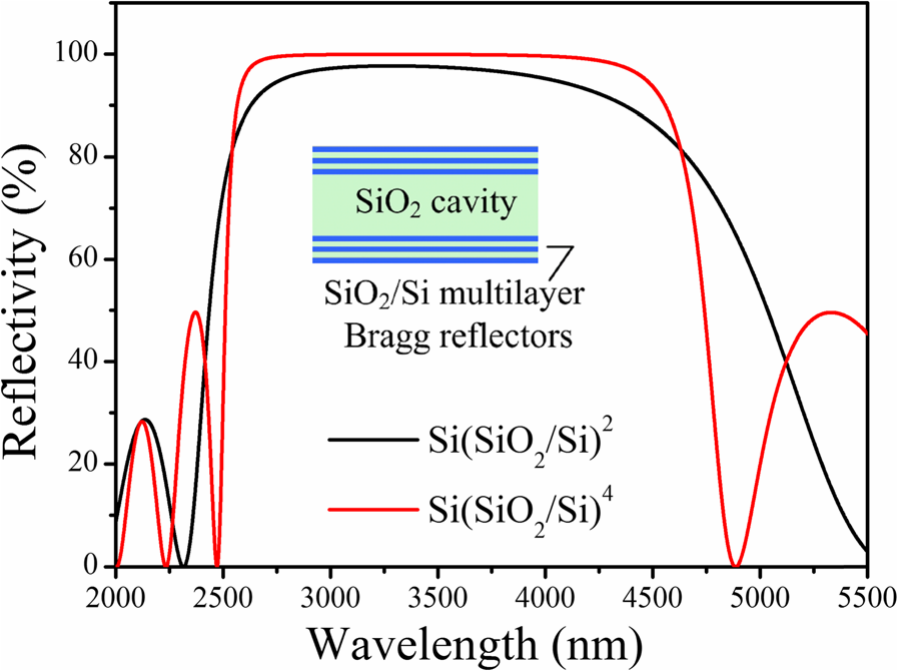
Comparison of simulated reflective spectra of Bragg reflectors with 2 pairs and 4 pairs of Si/SiO_2_ layers

Based on Eqs. (6, 7, 8), the reflectivity of Bragg reflectors can be calculated using MATLAB® software. The optimal designed thickness of Si/SiO_2_ layers can be referred from Table 1. Figure 2 shows a comparison of simulated reflectivity of Bragg reflectors with 2 pairs and 4 pairs of Si/SiO_2_ layers. It can be seen that the 4 pair structure has slightly higher reflectivity as well as sharper cutoff edge of reflective band in comparison with 2 pair structure, and the 4 pair structure also exhibits more out-of-band transmission-orders than 2 pair structure. From Fig. 2, Bragg reflector using 2 pairs of Si/SiO_2_ layers has wider reflective band capable of achieving the coverage of MIR band of 2.3~5 μm.

**Table 1:**
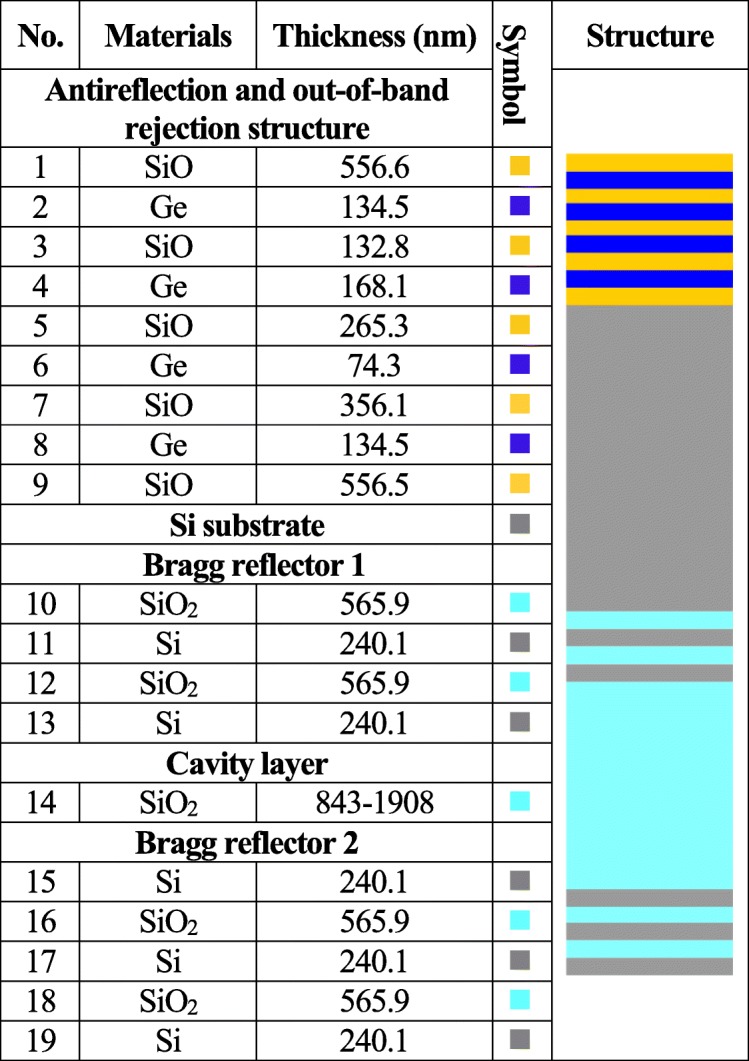
Fabrication parameters of LVOF

**Fig. 3:**
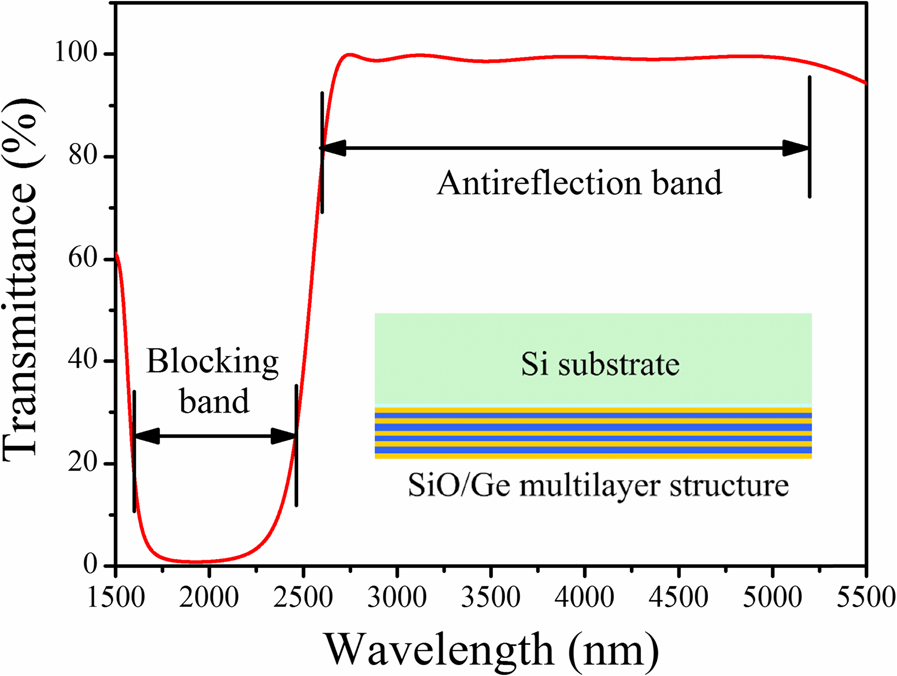
Simulated transmission spectrum of Ge/SiO multi-layer structure with both functions of the out-of-band rejection in 1.6~2.5 μm and the antireflection in 2.5~5 μm

In order to eliminate the influence of out-of-band transmission-orders of LVOF, an out-of-band blocking filter is generally used to reject other out-of-band transmission-orders of the LVOF. As shown in reference [22], an out-of-band blocking filter using multi-layer Si/SiO_2_ structure was placed on top of tapered Bragg reflector. In this work, we designed a full dielectric multi-layer structure on backside of Si substrate to achieve both functions of antireflection and out-of-band rejection in one. Considering the requirements of high infrared transparency and excellent mechanical strength, the Ge/SiO multi-layer structure was chosen to fabricate the antireflective film. Ge has a high refractive index of 4.2 and a high transmissivity in IR band of 1.7~23 μm while SiO has a low refractive index of 1.9 and a high transmissivity in IR band of 0.4~9 μm. Figure 3 shows the simulated transmittance spectra of Ge/SiO multi-layer structure with both functions of out-of-band blocking and antireflection. It is noticed that the thickness of each layer of Ge/SiO multi-layers is also referred from Table 1. It can be found that the multi-layer structure has a clear blocking band in the wavelength range of 1.6~2.5 μm, which can effectively suppress the transmission-orders of the LVOF in short wavelength region. At the same time, with Si as incident medium, the multi-layer structure also exhibits a perfect antireflective band in 2.5~5 μm with average transmittance of no less than 0.95.

**Fig. 4:**
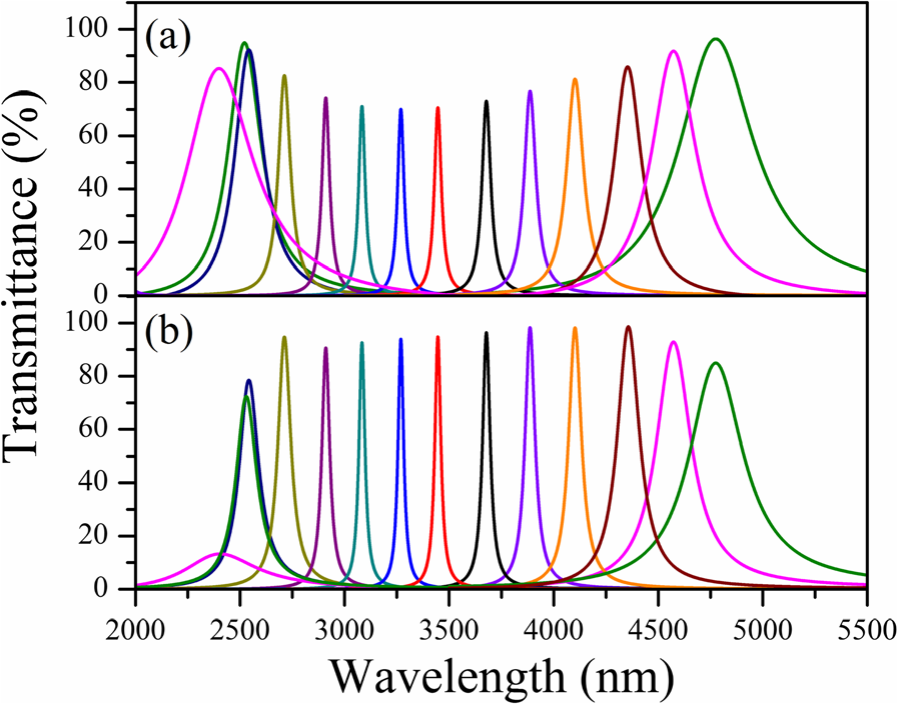
Comparison of simulated transmittance spectra of LVOF without (**a**) and with (**b**) Ge/SiO multi-layer structure

According to the absorption peaks of targeted gases, the thickness of SiO_2_ cavity was designed to vary linearly from 843 to 1908 nm, and 12 filter channels were specially designed, corresponding to the center wavelength from 2.55 to 4.80 nm. Figure 4 shows a comparison of simulated transmission spectra of LVOF without and with Ge/SiO multi-layer structure. It can be seen from Fig. 4 that the LVOF with Ge/SiO multi-layer structure exhibits narrower full-width-at-half-maximum (FWHM) in each transmission peak than that without Ge/SiO multi-layer structure. Apart from the transmissivity reduction in designed peaks of *λ*_*p*_ = 2.55 μm and *λ*_*p*_ = 4.8 μm, the transmissivity of all other peaks is clearly enhanced when using Ge/SiO multi-layer structure. Furthermore, it is found that both peaks in 4.60 μm and 4.80 μm have themselves corresponding common-mode in short wavelength region, e.g., *λ*_4.6_ = 2.36 μm and *λ*_4.8_ = 2.5 μm (see Fig. 4(a)), which can be explained by Eq.(10) when using different *k* values in same thickness of F-P cavity. Owing to the design of blocking band in short wavelength region, the peak in 2.36 μm was significantly weaken, as shown in Fig. 4(b).

**Fig. 5:**
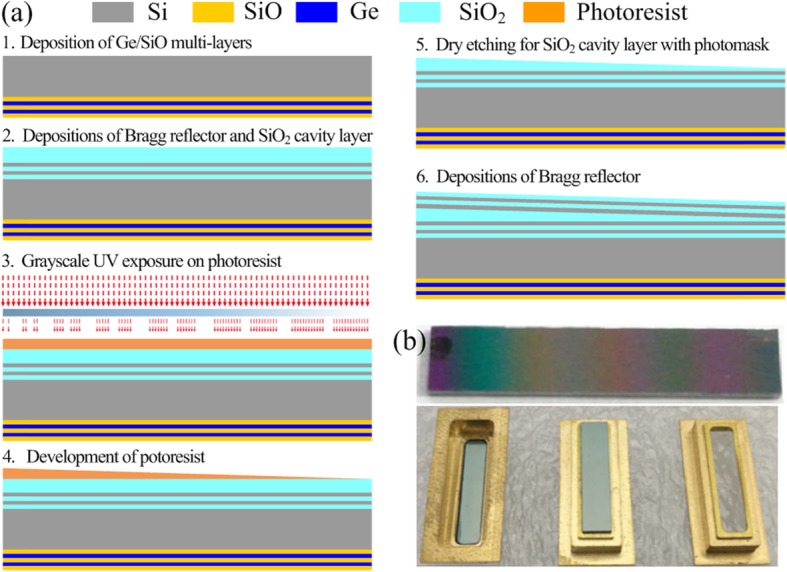
**a** MEMS-based fabrication process flow. b Photographs of actual LVOF and its package structure

**Fig. 6:**
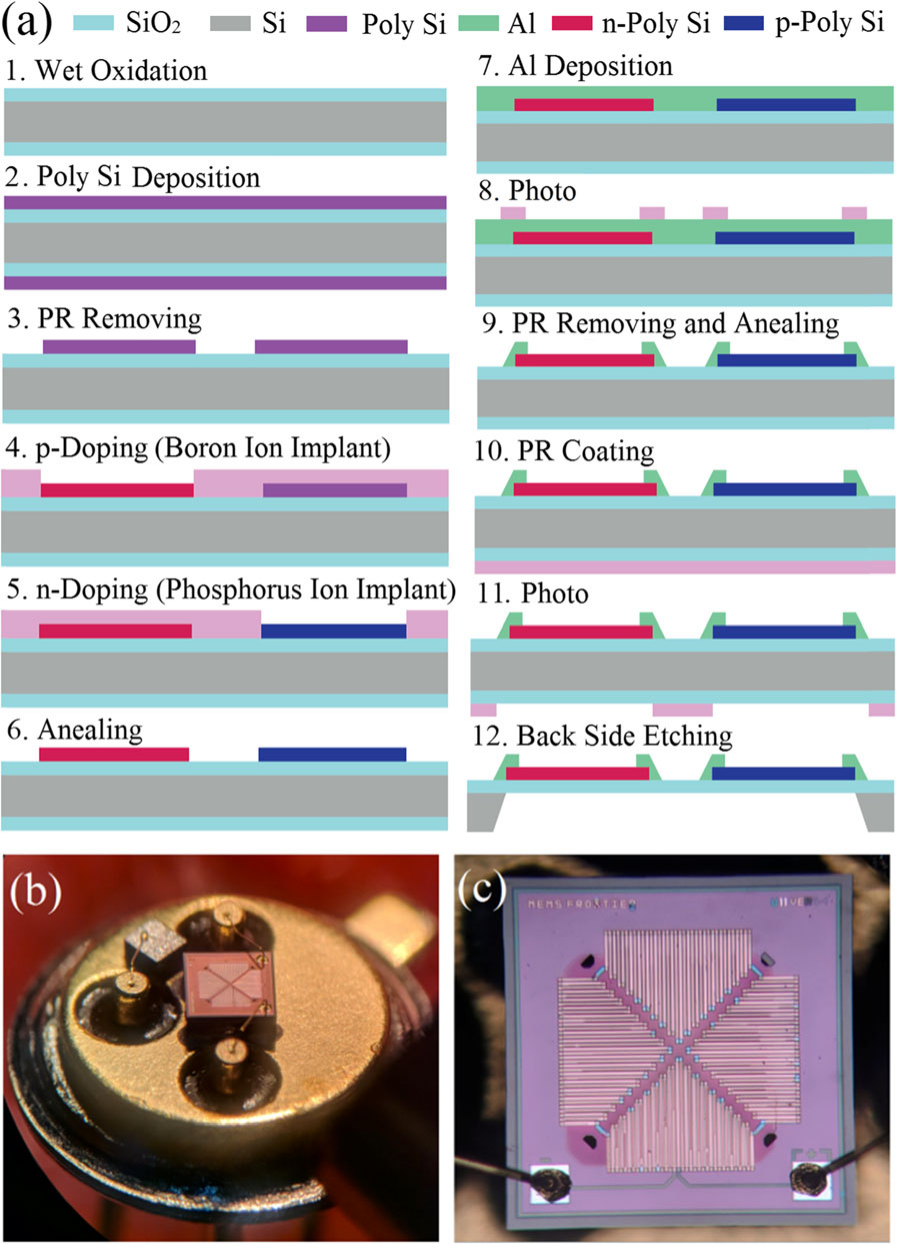
**a** Fabrication process flow of MEMS-based thermopile detector. **b** Photographs of MEMS-based thermopile chip packaged in socket. **c** The enlarged view of thermopile chip

Figure 5 a shows the fabrication process flow of LVOF. The fabrication parameters of LVOF follow the optimal designed parameters, as presented in Table 1. Firstly, Ge/SiO multi-layer structure was deposited on the backside of silicon wafer (see Fig. 5a-1). Next, the Si/SiO_2_ multi-layer structure was deposited on the front side of silicon wafer to form the Bragg reflector 1, and then the SiO_2_ cavity layer was deposited on Bragg reflector 1 (see Fig. 5a-2). The third step was to evenly spin-coat photoresist on the cavity layer, and then a special gray scale photomask with linear change in UV transmission intensity from low (dark) to high (light) along the length of LVOF was used to expose the photoresist (see Fig. 5a-3). Such special photomask could make the cross-linked thickness of resist to have a linear change along the length of LVOF. The fourth step was to develop the photoresist to form a wedge-shaped structure, and then a hot reflow process was used to smooth the surface of wedge-shaped structure (see Fig. 5a-4). Next, the tapered photoresist structure was transferred to the underlying SiO_2_ cavity layer by dry etching (see Fig. 5a-5). Finally, Bragg reflector 2 with the Si/SiO_2_ multi-layers was deposited on the tapered cavity layer (see Fig. 5a-6). Figure 5 b shows the photographs of the actual LVOF and its package structure.

### Design and Fabrication of IR Thermopile Detectors

Thermopile detectors have several advantages for the application of IR gas detecting. Firstly, it does not need a power supply, and thus it rejects the noise voltage against the power source. Secondly, because the current flowing through the thermopile detector is very small, a low-frequency noise (1/f noise) caused by the driving current can also be ignored. Finally, the thermopile detectors can be used without any chopper to detect infrared DC and AC radiation [24]. By contrast, the pyroelectric IR detectors have higher responsivity and signal-to-noise ratio (SNR) than thermopile detectors, but they require a chopper to detect the incident radiation. This will result in the increase of detector size as well as the application cost. Therefore, the thermopile detectors are more suitable for the application of the low-cost and miniaturized gas detectors.

**Fig. 7:**
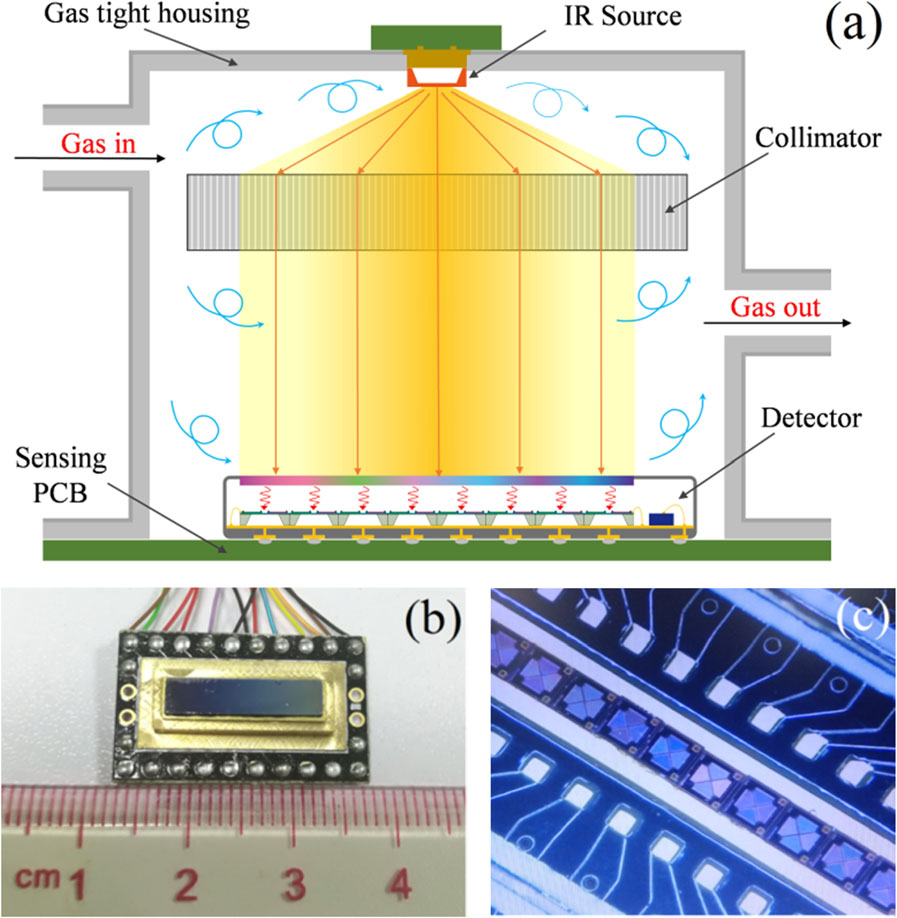
**a** Schematic diagram of working principle of miniaturized mixed gas detector. **b** Photographs of the miniaturized LVOF-based spectrometer. **c** The thermopile chip array packaged in socket

**Fig. 8:**
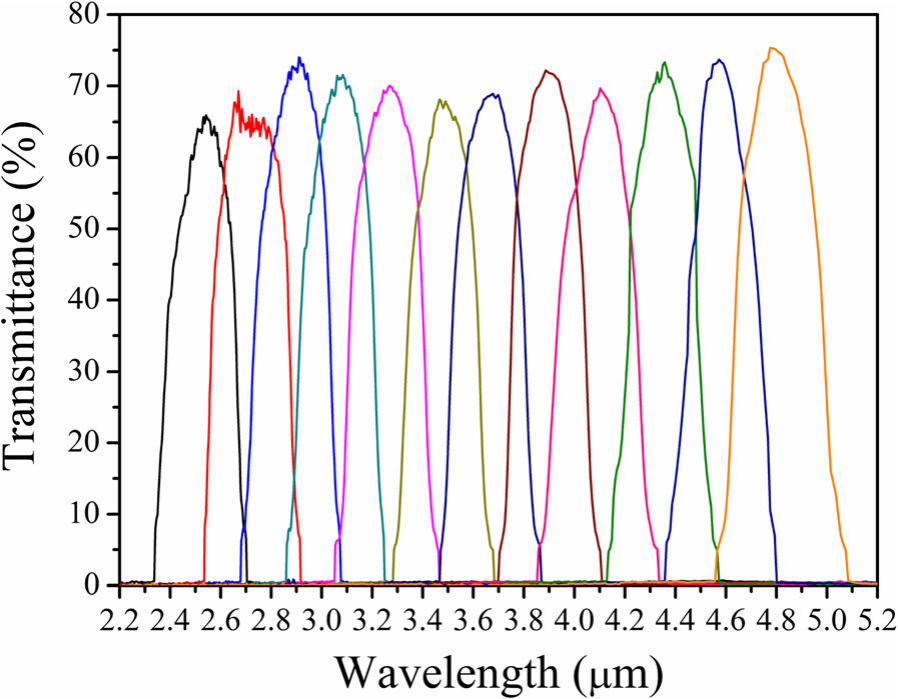
Measured spectral response of LVOF

**Fig. 9:**
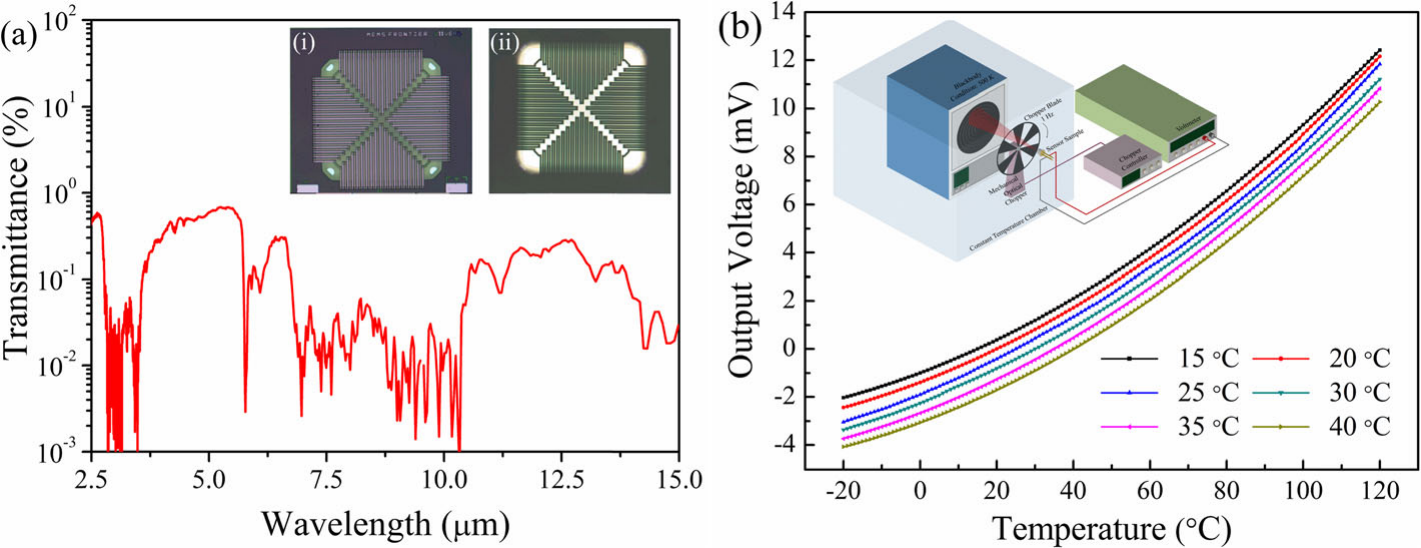
**a** Spectral response of thermopile detector. Inset is optical micrographs of the (i) front-side and (ii) backside of the thermopile chip. **b** Thermoelectric characteristics of thermopile detector at different ambient temperatures. Inset is the schematic diagram of measurement setup

**Fig. 10:**
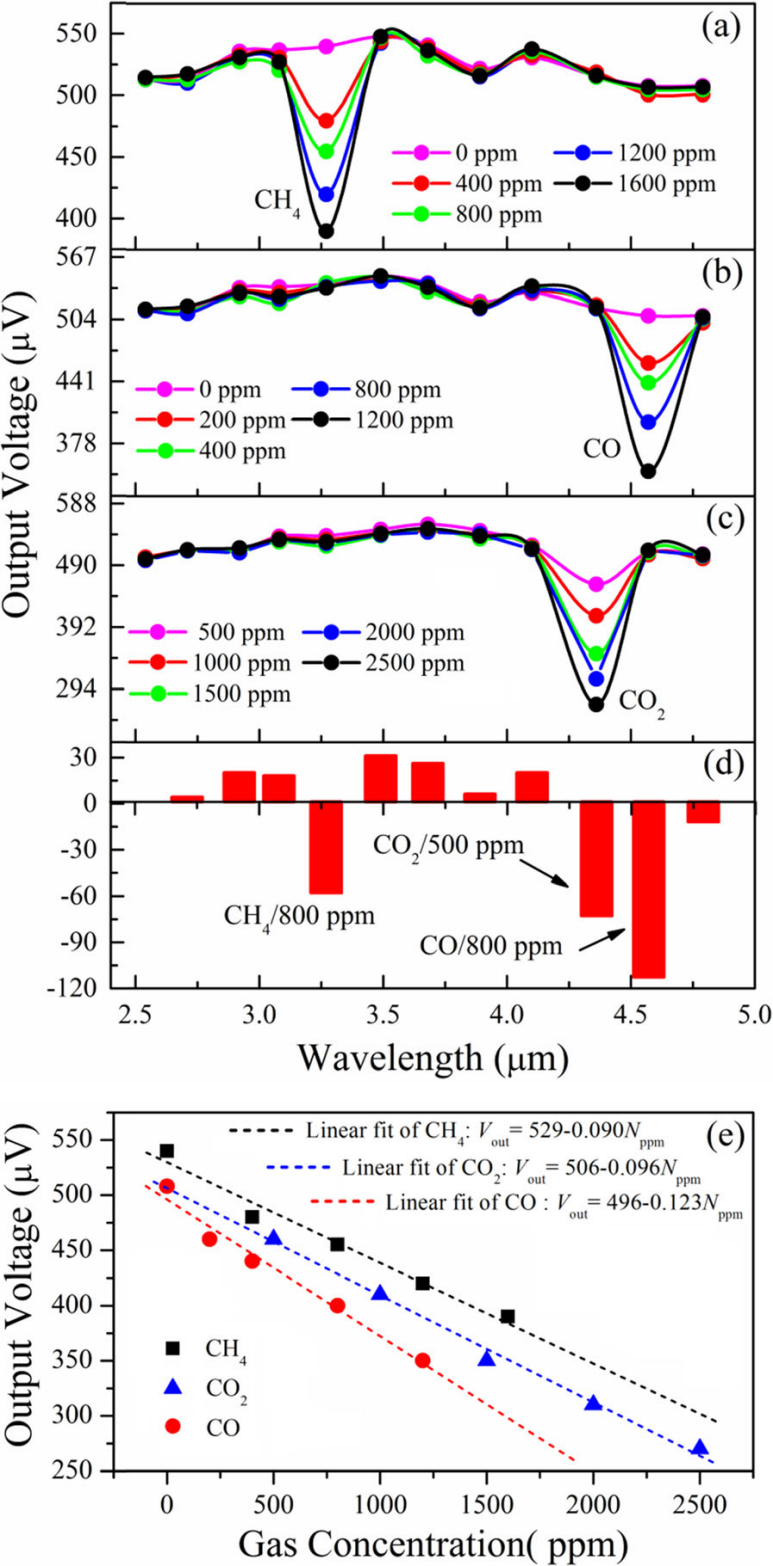
Spectral response of CH_4_ (**a**), CO (**b**), and CO_2_ (**c**) at different concentrations; Spectral response histogram of mixed gases based on CH_4_/800 ppm, CO_2_/500 ppm, and CO/800 ppm (**d**); Linear dependence of output voltage on gas concentration (**e**)

In this work, the thermopile detector was designed to generate the amplified Seebeck voltage by connecting multiple pairs of thermocouple elements in series to form a compact structure. The size of thermopile chip is designed as 1.1 mm (length) × 1.1 mm (width) with the active size of 0.35 mm × 0.35 mm. Figure 6 a shows the fabrication process flow of MEMS-based thermopile detector. Firstly, thermal oxidation technology was used to generate SiO_2_ layer with thickness of 0.6 μm at the silicon wafer (see Fig. 6a-1), and then poly-silicon (poly-Si) with thickness of 0.5 μm was deposited on SiO_2_ layer (see Fig. 6a-2). Next, the poly-Si was structured to form the thermocouple beams by the lithographic and RIE techniques (see Fig. 6a-3). Following above step, the boron was implanted with 45 keV and 5.5 × 10^15^ cm^−2^ to realize *p*-type poly-Si and phosphorous was implanted with 40 keV and 7 × 10^15^ cm^−2^ to realize *n*-type poly-Si (see Fig. 6a-4 and -5), and then a post-annealing (see Fig. 6a-6) was conducted at 1000 °C for 30 min. In next step, aluminum (Al) film was deposited and patterned on the top of device layer to define electric connection of thermocouples and bonding pads (see Fig. 6a-7 and -8), and then a metallization annealing process at 400 °C for 30 min was performed for realizing the ohmic contacts between the doped poly-Si and the Al (see Fig. 6a-9). Finally, the active membrane was formed using the silicon etching process using DIRE from backside of silicon wafer (see Fig. 6a-10, -11, and -12). Figure 6 b shows the photographs of MEMS-based thermopile chip packaged in socket, and Fig. 6 c exhibits the enlarged view of thermopile chip.

### Design and Fabrication of Miniaturized Mixed Gas Detectors

Figure 7 a shows the schematic diagram of working principle of mixed gas detector. The mixed gas detector consists of an IR source, a collimator, a gas cell, and an integrated LVOF-based spectrometer. The IR light emitted by IR light source was aligned by the collimator and then was incident on the LVOF. As a result, the continuous IR spectrum was transform into multiple discrete narrow band-pass spectra, separately corresponding to each filter channel with peak wavelength in linear variation. A linear array of thermopile detectors was placed under the LVOF to transfer the incident light energy from different filter channels into electric signal. The compact integration of the LVOF and the thermopile detector array makes a miniaturized LVOF-based spectrometer. The miniaturized mixed gas detectors have significant advantages in reducing the overall size of multi-gas detectors as well as decreasing the fabrication cost and power consumption by using a light source, a gas cell, and a data process element when compared with the traditional NDIR gas detectors.

Figure 7 b and c show the photographs of the miniaturized LVOF-based spectrometer and the thermopile chip array packaged in socket, respectively. A total of 12 thermopile chips were integrated as a linear array and installed side by side in socket, above which is the LVOF window. Such design will operate IR wavelength from 2.3 to 5.0 μm, with an excellent linear dependence of ~ 156 nm/mm over 16 mm. The concentration of each gas in gas mixture can be detected separately by controlling a switch array to sweep-read and process data from each thermopile chip.

## Results and Discussion

To measure the optical response of fabricated LVOF, the LVOF should be scanned through its length direction at every position point of filter channels. A micro-spot test method was used to obtain the transmission spectra of LVOF by using a commercial FTIR spectrometer. The LVOF was placed in a sample fixture and moved passing a slit plate with an optical aperture of 350 μm. The sampling spots were taken at intervals of 1.1 mm (width of thermopile detector) from the starting position at 1.25 mm along the length of LVOF. A total of 12 sampling points were measured to cove MIR wavelength range from 2.3 to 5.0 μm. For each spectrum, 50 scans were averaged to increase the SNR. Figure 8 shows the spectral response of LVOF. It can be seen that the wavelength of transmission peaks follows the linear change with the change of test position. The mean FWHM of LVOF is ~ 400 nm, and mean transmittance of peak gets close to ~ 70% with cut-off transmittance of ≤ 0.5%.

The spectral response of thermopile detector, as shown in Fig. 9a, was measured using same measurement method and setup as that of LVOF. It can be seen that the active membrane (see the inset of Fig. 9a) has a very low transmittance (≤ 1.0%) in 2.5~15 μm. This means that IR energy in this waveband can be fully absorbed and transferred into thermal energy by the heavy doped poly-Si. The thermopile detectors were characterized through a measurement setup consisting of IR source, voltmeter, chopper, and constant temperature chamber (see the inset of Fig. 9b). A standard blackbody was used as the IR source to calibrate the detector, and the temperature of blackbody can be controlled accurately according to the requirement of measurement. Figure 9 b shows the thermal-electric characteristics of thermopile detectors under different ambient temperatures. It is demonstrated that the thermopile detectors have a high responsivity of 146 μV/°C (*T*_Blackbody_ = 100 °C) at the condition of room temperature.

In order to verify gas analysis capacity of mixed gas detectors, some standard gases with strong and wide absorption peaks were selected as the measured gases. The characteristic absorption peaks of gases used in our experiment are CH_4_/~ 3.3 μm, CO_2_/~ 4.3 μm, and CO/~ 4.6 μm, respectively. The single gas at different concentrations and the mixed gas at different mixing ratios were measured, respectively. The gas flows getting in and out gas housing were controlled through the mass flowmeter, and some commercial standard gas detectors were used to calibrate the gas concentrations.

Figure 10 shows the spectral responses of three kinds of gases and their mixture at different concentrations. The IR-enhanced absorptions are found in 5th (see Fig. 10(a)), 11th (see Fig. 10(b)), and 10th (see Fig. 10(c)) filter channel, corresponding to characteristic absorption peaks of CH_4_, CO, and CO_2_, respectively. Figure 10 (e) exhibits the dependence of output voltage on gas concentration. By means of best linear fitting for the experimental data of CH_4_, CO_2_, and CO, the fitting equations were obtained. The coefficient of determination (*R*^2^), which is commonly used as a goodness-of-fit, reaches 0.968, 0.991, and 0.969 for CH_4_, CO_2_, and CO, respectively. It is seen that the output voltage linearly changes with the change of gas concentrations. It has been measured that the sensitivity for CH_4_, CO_2_, and CO is − 0.090 μV/ppm, − 0.096 μV/ppm, and − 0.123 μV/ppm, respectively. According to current structure and device parameters, the range of gas detection is about 50~3000 ppm. Next, the mixed gas based on the concentration of CH_4_/800 ppm, CO_2_/500 ppm, and CO/800 ppm was measured. By normalizing output voltage to reference voltage of filter channel at center wavelength of 2.55 μm, three obvious spectral absorption columns corresponding the signatures of CH_4_, CO_2_, and CO are found in the histogram of spectral response (see Fig. 5d), which verifies the application feasibility of mixed gas detecting. It is noted that in current design structure of gas cell, the short light-path length and low array pixel limit the minimum concentration of gas detection as well as the number of gases that could be measured. Meanwhile, some gases with fine structure in the absorption peaks also cannot be identified.

## Conclusion

In conclusion, the design, fabrication, and characterization of a MIR LVOF and a MEMS-based infrared thermopile detector were presented, respectively. The LVOF was designed as a linear array of F-P type resonators to transform MIR continuous spectrum into multiple narrow band-pass spectra, separately corresponding to each filter channel with peak wavelength in linear variation. A Si/SiO_2_ multi-layer structure was used to fabricate the Bragg reflectors on the both sides of SiO_2_ tapered cavity, and a Ge/SiO multi-layer structure on the backside of Si substrate was used to achieve both functions of antireflection and out-of-band rejection. The MEMS-based thermopile detector was designed and fabricated to generate the amplified Seebeck voltage by connecting multiple pairs of *p*- and *n*-poly-Si/Al thermocouple elements in series to form a compact structure. The LVOF was installed above a linear array of MEMS-based thermopile detectors to form a miniaturized MIR spectrometer, which can be used to detect mixed gases and was experimentally verified by the quantification and identification of CH_4_/CO_2_/CO mixed gases.

## Data Availability

All data generated or analyzed during this study are included in this published article.

## Abbreviations

LVOF: Linear variable optical filter
NDIR: Non-dispersive infrared
F-P: Fabry-Pérot
NIR: Near-IR
MIR: Middle-IR
FTIR: Fourier transform infrared
GC: Gas chromatography
MEMS: Micro-Electro Mechanical Systems
MOSs: Metal-oxide semiconductors
CNTs: Carbon nanotubes
N_2_O: Nitrogen oxides
CO_2_: Carbon dioxide
CO: Carbon monoxide
CH_4_: Methane
FWHM: Full-width-at-half-maximum
SNR: Signal-to-noise ratio
